# Supplementary material for: A Predictive Processing Model of Perception and Action for Self-Other Distinction
Source: Front Psychol. 2018 Dec 3;9:2421. doi: 10.3389/fpsyg.2018.02421 (PMC6287016; doi:10.3389/fpsyg.2018.02421)
Supplement: Supplementary file 1 [file Data_Sheet_1.PDF]

Supplementary Material: A predictive processing model of perception and action for self-other distinction

Sebastian Kahl and Stefan Kopp
*Correspondence:
Author Name: Sebastian Kahl
skahl@uni-bielefeld.de

1 DOMAIN AND DYNAMICAL SYSTEM

Our hierarchical generative model can be expressed in terms of functions that map the internal state space from one level L_X in the hierarchy to the domain of it's next lower level L_{X-1}, i.e., $L_{X-1} = l(L_X)$, with l as a placeholder for a specific mapping. The lowest level in the hierarchy will then map to the environmental state space φ.

In the highest level **Schemas** map to it's next lower level **Sequences** with $s : C \mapsto S$ where C cluster Sequences.

Level **Sequences** maps to it's next lower level **Vision** with $v : S \mapsto V$ where S consists of sequences of states from V. Then similarly, Sequences maps to the Motor level M using function $m : S \mapsto M$.

The lowest levels of the hierarchy are described by M (**Motor**) and V (**Vision**). Here, only the Motor level can directly influence the environmental state space through $m : M \mapsto \vartheta$ where M represents movements in ϑ.

We define the environmental state space in terms of a dynamical system of $\Omega = (X, T, \varphi, \vartheta)$, with space $X = \mathbb{R}^2$ and discrete time $T = \mathbb{N}$. $\varphi : X \times T \to X$ is a function of discrete movements over time and $\vartheta : T \to X$ is a function of movements in the state space (updating positions).

As an extension, our hierarchical generative model can be described as *active* in the sense that each level maps external (L_e) to internal states (L_i) to minimize entropy. What is described as external depends on where in the hierarchy the level is situated. In the lowest level, i.e., Motor and Vision levels, the external states describe the sensory states of the system. Entropy minimization can be described as a function of $h(L_e, L_i)$ which maps external states onto internal states in a way that minimizes entropy (see eq. 1).

$$\begin{aligned} h(L_e, L_i) &= \arg\min_{l \in L_i} H(L_e|l) \\ H(L_e|L_i) &= -\sum_{l \in L_e} p(l|L_i)\; ln\, p(l|L_i). \end{aligned} \tag{1}$$

2 HIERARCHICAL GENERATIVE MODEL

In the following the specific levels of the generative model (C, S, M, V, x) are described (see joint distribution in eq. 2). These are sequentially updated, i.e., they are updated in sequence updated from it's

next higher and next lower levels, learning to represent and produce the states in the next lower level and the environment with $x = \varphi(x, t)$.

$$P(C, S, M, V, x) = P(C) \cdot P(S|C) \cdot P(V|S) \cdot P(M|S) \cdot P(x|M) \tag{2}$$

2.1 Schema level

The Schema level $L_C = (C, F_C, \pi_C)$ (see eq. 3) contains a discrete probability distribution over discrete states $C = \{c_1, \ldots, c_n\}$, the free energy over the probability distribution F_C and the precision over the probability distribution π_C, calculated as the reciprocal of the variance σ^2 over $P(C)$. Each schema c_i clusters similar sequences $\mathbb{S} \subseteq S$ from Sequence level L_S, and the schema's prototype c_i^η over it's sequences.

$$\begin{aligned}
C &= \{c_i, \ldots, c_n\} \\
F_C &= F(C) \\
\pi_C &= \tfrac{1}{\sigma^2(P(C))} \\
c_i &= (\mathbb{S}, c_i^\eta) \\
D_\iota^{n\times n} &= \begin{bmatrix} \iota(s_1, s_1) & \ldots & \iota(s_n, s_1) \\ \vdots & \vdots & \vdots \\ \iota(s_n, s_1) & \ldots & \iota(s_n, s_n) \end{bmatrix} \Bigg| \forall s_{i,j} \in \mathbb{S} \\
c_i^\eta &= s_j \mid j = \arg\min_k \sum_i^k (D_\iota)_{i,k}
\end{aligned} \tag{3}$$

The schema prototype c_i^η is calculated over the distance matrix over all it's sequences $D_\iota^{n\times n}$ (for distance function ι see eq. 6), finding the sequence with the minimal summed distance to all other sequences. This effectively defines the *median* sequence clustered into the schema as the schema's prototype (similar as in *k-medoid clustering*).

The generative model (eq. 4) calculates it's posterior using the soft evidence „all things considered" method over sequences given the schema *cluster* it belongs to (for more information, please see (Darwiche, 2009, chapter 3.6.1)).

$$P_{bu}(C) = P_t(C) = \sum_{s_i \in S} P_{t-1}(s_i) \cdot P_t(c_i|s_j) \mid c_j \in C \tag{4}$$

2.2 Sequence level

The Sequence level $L_S = (S, F_S, \pi_S)$ (see eq. 5) contains a discrete probability distribution over discrete states $S = \{s_1, \ldots, s_m\}$, the free energy over the probability distribution F_S and the precision over the probability distribution π_S. Each sequence s_i contains a tuple of observed movements $(o_1, \ldots, o_k)$ in polar cooridnates at time $t \in T$ with $o_i = (\theta, r)$, and the time delay between observations $(\Delta_2, \ldots, \Delta_k)$, with

$\Delta_i t = t_i - t_{i-1}$.

$$\begin{aligned} F_S &= F(S) \\ \pi_S &= \frac{1}{\sigma^2(P(S))} \\ S &= \{s_1, \ldots, s_m\} \\ s_i &= ((o_1, \ldots, o_t), (\Delta_2, \ldots, \Delta_t)) \end{aligned} \tag{5}$$

The difference between sequences $\iota(s_i, s_j)$ is calculated using the so-called „alphabetic Jensen-Shannon Distance" (aJSD, Mateos et al. (2017)), which first discretizes timeseries data in a combined probability space, on which then the Jensen-Shannon Distance can be applied (see the algorithm description in eq. 6). First, both s_i and s_j are converted into binary sequences b_i and b_j, which are then both split into consecutive words $w_t(b)$ of word length $d = 3$ with step size $\tau = 1$ and collected in alphabets of words W_i and W_j. Then, probability distributions P^{W_i} and P^{W_j} are formed from word frequencies for each alphabet. The Jensen-Shannon Distance D_{JS} is calculated over these word frequency probability distributions.

$$\begin{aligned} \varrho_t &= \begin{cases} 1, & \text{if } o_{t+1} > o_t \\ 0, & \text{if } o_{t+1} \leq o_t \end{cases} \Bigg| \forall o_{t+1}, o_t \in s_l \\ b_l &= (\varrho_1, \ldots, \varrho_t) \\ w_t^{(d,\tau)}(b_l) &= (\varrho_{t-(d-1)\tau}, \ldots, \varrho_t) \mid t \geq (d-1)\tau, \varrho_t \in b_l \\ W^{(d,\tau)}(b_l) &= \{w_1^{(d,\tau)}, \ldots, w_t^{(d,\tau)}\} \\ P^{W_l} &= P(W_l^{(d,\tau)}) \\ \iota(s_i, s_j) &= D_{JS}(P^{W_i} || P^{W_j}) \end{aligned} \tag{6}$$

We have two posterior distributions in the generative model for L_S resulting from a bottom-up and a top-down update process ($P_{bu}(S)$, and $P_{td}(S)$ respectively). The top-down update for $P_{td}(S)$ calculates the posterior from a mixture of experts in Schema level L_C (see eq. 7).

$$P_{td}(S) = \sum_{c \in C} P(S|c) \cdot P(c) \tag{7}$$

For the bottom-up update we need to calculate the sequence probability given observations represented in Vision level L_V. L_V represents only singular observations of movement, so in order to calculate their likelihood we collect observations in a temporally growing sequence $s' = (o'_1, \ldots, o'_\tau)$. We calculate the likelihood for each sequence $P(s'|s_i)$ in which the sequence difference ι is weighted by an exponential factor, which calculates the temporal precision of the observed state. This is in effect comparable to calculating a joint probability for all observation events $P(o'_1, \ldots, o'_\tau|s_i)$. Calculating the posterior then simply is a Bayesian inversion (eq. 9).

$$P(s'|s_i) = \iota(s', s_i) \cdot e^{-\frac{(s'_{\Delta t} - s_{i,\Delta t})^2}{2\,\pi_S^2}} \tag{8}$$

$$P_{bu}(S) \approx P(S|o_1, \ldots, o_\tau) = \frac{P(o_1, \ldots, o_\tau|S)\; P(S)}{P(o_1, \ldots, o_\tau)} \tag{9}$$

Since a sequence will be of no use as a prediction for Vision level L_V we need to obtain the next observation probability from the predicted sequence (if there is any) given the predicted sequence and prior observations (see eq. 10). The resulting distribution is compatible with representations of Motor level L_M and is used accordingly as $P(M|S)$.

$$P(V|S) \approx P(o_{\tau+1}|o'_1, ..., o'_\tau, s_i) = \frac{P(o'_1, ..., o'_\tau, o_{\tau+1}|s_i)}{P(o'_1, ..., o'_\tau|s_i)} \Bigg| \forall o_{\tau+1} \in V \tag{10}$$

2.3 Vision level

The Vision level $L_V = (V, F_V, \pi_V)$ (see eq. 11) contains a discrete probability distribution over discrete states $V = \{v_1, \ldots, v_i\}$ representing perceived movement angles, the free energy over the probability distribution F_V and the precision over the probability distribution π_V. Each v_i represents a movement perceivable by the model.

$$\begin{aligned} V &= \{v_1, \ldots, v_i\} \\ F_V &= F(V) \\ \pi_V &= \tfrac{1}{\sigma^2(P(V))} \end{aligned} \tag{11}$$

The level calculates a top-down updated posterior P_{td} (see eq. 12) and a bottom-up updated posterior P_{bu} (see eq. 13). P_{td} is updated from a mixture of experts in Sequence level L_S.

$$P_{td}(V) = \sum_{s \in S} P(V|s) \cdot P(s) \tag{12}$$

The bottom-up update is a mapping from input state space $\varphi(x, t)$ to the model's movement reportoire using a gaussian likelihood function for each available movement $v_i \in V$, given $\sigma = 0.1$.

$$\begin{aligned} P(\varphi(x,t)|v_i) &= e^{-\frac{(\varphi(x,t)-v_i)^2}{2\sigma^2}} \\ P_{bu}(V) \approx P(V|\varphi(x,t)) &\propto P(\varphi(x,t)|V)\ P(V) \end{aligned} \tag{13}$$

2.3.1 Salience detector

To detect a salient input event we calculate the model free energy given two consecutive input events from state space $\varphi(x, t)$ Vision level V. If $F_t(V) > F_{t-1}(V)$ the updated model was not able to correctly predict the current input event. This is a salient event which will be communicated to the next higher level L_S.

2.3.2 Oculocentric coordinates in Vision

Salient movements observed in the environment are mapped to an oculocentric coordinate system before they are sent to the next higher level L_S. This way we map salient events in the environment to internal representations that are reusable as low-level action goals for Vision and Motor levels. The oculocentric coordinates are relative polar coordinates (ϕ, r) of the visual field, which when seen in sequence are similar to saccadic eye movements. In Motor level L_M these coordinates will guide action in the form of movement goals.

2.4 Motor level

The Motor level $L_M = (M, F_M, \pi_M)$ (see eq. 11) contains a discrete probability distribution over discrete states $M = \{m_1, \ldots, m_i\}$ representing movement angles, the free energy over the probability distribution M_F and the precision over the probability distribution π_M. Each m_i represents a movement perceivable by the model.

$$\begin{aligned} M &= \{m_1, \ldots, m_i\} \\ F_M &= F(M) \\ \pi_M &= \tfrac{1}{\sigma^2(P(M))} \end{aligned} \tag{14}$$

The level again calculates a top-down updated posterior P_{td} (see eq. 15) and a bottom-up updated posterior P_{bu} (see eq. 16). P_{td} is updated from a mixture of experts in Sequence level L_S.

$$P_{td}(M) = \sum_{s \in S} P(M|s) \cdot P(s) \tag{15}$$

The bottom-up update is a mapping from input state space $\varphi(x, t)$ to the model's movement reportoire using a gaussian likelihood function for each available movement, given $\sigma = 0.1$.

$$\begin{aligned} P(\varphi|m_i) &= e^{-\frac{(\varphi(x,t)-m_i)^2}{2\sigma^2}} \\ P_{bu}(M) &\approx P(M|\varphi(x,t)) \propto P(\varphi(x,t)|M)\ P(M) \end{aligned} \tag{16}$$

2.4.1 Motor coordination in active inference

To allow for smooth and curving trajectories that are similar to handwriting in spatial and temporal properties, we were inspired by work on dynamic movement primitives (DMP) that are used for modeling attractor behaviors of autonomous nonlinear dynamical systems with the help of statistical learning techniques Ijspeert et al. (2013). We will not make use of the DMPs ability to learn and reproduce trajectories, but will configure a damped spring system similarly to a DMP and instead of applying a forcing term f that activates the system's nonlinear dynamics over time we make use of an obstacle avoidance technique mentioned in Hoffmann et al. (2009) which we adpoted and inverted its force to actually move towards the goal in a goal forcing function g (see eq. 17). The reason for this is that when we simply applied the spring system to each goal sequentially, we would accelerate toward and slow down at the goal. To keep up the momentum we need to look ahead several goals x_{i+3} (here 3 steps ahead) in the core spring system, but with a goal forcing function that sequentially tries to visit each goal x_i. α, β, γ and μ are constants that specify the behavior of the system. φ is the angle to the goal (or its velocity) and y is the current position.

$$\begin{aligned} \varphi &= \varphi_{x_i} - \varphi_{\dot{y}} \\ \dot{\varphi} &= \gamma\ \varphi\ e^{-\mu|\varphi|} \\ g &= (x_i - y)\ \dot{\varphi} \\ \ddot{y} &= \alpha(\beta(x_{i+3} - y) - \dot{y}) + g \end{aligned} \tag{17}$$

The resulting acceleration $\ddot{y}$ will be twice integrated before it is applied as an environmental state space position $\vartheta(t) = y$.

Once the goal is reached the Motor level L_M will send a direct signal to the Vision level L_V, where the current location will be evaluated, given the goal location that was also received by L_V.

REFERENCES

Darwiche, A. (2009). *Modeling and reasoning with Bayesian networks* (Cambridge university press)

Hoffmann, H., Pastor, P., Park, D.-H., and Schaal, S. (2009). Biologically-inspired dynamical systems for movement generation: Automatic real-time goal adaptation and obstacle avoidance. In *2009 IEEE International Conference on Robotics and Automation (ICRA)* (IEEE), 2587–2592

Ijspeert, A. J., Nakanishi, J., Hoffmann, H., Pastor, P., and Schaal, S. (2013). Dynamical movement primitives: learning attractor models for motor behaviors. *MIT Press* 25, 328–373

Mateos, D. M., Riveaud, L. E., and Lamberti, P. W. (2017). Detecting dynamical changes in time series by using the Jensen Shannon divergence. *Chaos: An Interdisciplinary Journal of Nonlinear Science* 27, 083118–14
